# Fabrication, Characterization and Cellular Compatibility of Poly(Hydroxy Alkanoate) Composite Nanofibrous Scaffolds for Nerve Tissue Engineering

**DOI:** 10.1371/journal.pone.0057157

**Published:** 2013-02-27

**Authors:** Elahe Masaeli, Mohammad Morshed, Mohammad Hossein Nasr-Esfahani, Saeid Sadri, Janneke Hilderink, Aart van Apeldoorn, Clemens A. van Blitterswijk, Lorenzo Moroni

**Affiliations:** 1 Department of Textile Engineering, Isfahan University of Technology, Isfahan, Iran; 2 Department of Cell and Molecular Biology, Cell Science Research Center, Royan Institute for Biotechnology, ACECR, Isfahan, Iran; 3 Department of Tissue Regeneration, University of Twente, Enschede, The Netherlands; 4 Department of Electrical and Computer Engineering, Isfahan University of Technology, Isfahan, Iran; University of Leipzig, Germany

## Abstract

Tissue engineering techniques using a combination of polymeric scaffolds and cells represent a promising approach for nerve regeneration. We fabricated electrospun scaffolds by blending of Poly (3-hydroxybutyrate) (PHB) and Poly (3-hydroxy butyrate-co-3- hydroxyvalerate) (PHBV) in different compositions in order to investigate their potential for the regeneration of the myelinic membrane. The thermal properties of the nanofibrous blends was analyzed by differential scanning calorimetry (DSC), which indicated that the melting and glass temperatures, and crystallization degree of the blends decreased as the PHBV weight ratio increased. Raman spectroscopy also revealed that the full width at half height of the band centered at 1725 cm^−1^ can be used to estimate the crystalline degree of the electrospun meshes. Random and aligned nanofibrous scaffolds were also fabricated by electrospinning of PHB and PHBV with or without type I collagen. The influence of blend composition, fiber alignment and collagen incorporation on Schwann cell (SCs) organization and function was investigated. SCs attached and proliferated over all scaffolds formulations up to 14 days. SCs grown on aligned PHB/PHBV/collagen fibers exhibited a bipolar morphology that oriented along the fiber direction, while SCs grown on the randomly oriented fibers had a multipolar morphology. Incorporation of collagen within nanofibers increased SCs proliferation on day 14, GDNF gene expression on day 7 and NGF secretion on day 6. The results of this study demonstrate that aligned PHB/PHBV electrospun nanofibers could find potential use as scaffolds for nerve tissue engineering applications and that the presence of type I collagen in the nanofibers improves cell differentiation.

## Introduction

Neural injuries are very common in clinical practice and may lead to permanent disabilities in patients. Existing tissue engineering approaches focus on finding alternative procedures for nerve regeneration using polymeric scaffolds 1]. Various strategies have been employed to create a biodegradable nerve guidance scaffold to assist regenerating axons by serving as a growth substrate for neural cells, 2]. Highly porous electrospun nanofiber matrices are a logical choice because of the physical and structural similarities to the extracellular matrix (ECM) components such as collagen fibers and their high surface area 3]. Recent reports on neural regeneration also highlight the promise of using electrospun nanofibrous scaffolds in combination with mesenchymal stem cells (MSCs) 4], human adipose tissue-derived stem cells (hASCs) 5], nerve precursor cells (NPCs) 6], neural stem cells 7] or Schwann cells (SCs) 8]. Among the physical and chemical cues that can be imparted to improve neural regeneration, nanofiber orientation has been shown to increase ECM production. Fiber alignment greatly influenced cell growth and related functions in different cell sources such as neurons and human coronary artery smooth muscle cell (SMCs) 9,10]. As a result of contact guidance, a cell has the maximum probability of migration in preferred directions associated with chemical, structural and mechanical properties of the substrate 11]. It has been reported in different studies that, unidirectional aligned nanofibers can provide better contact guidance effects towards neurite outgrowth and help in providing cues to enhance SCs extension and axon regeneration 9,12–14].

SCs are the main glial cells of the peripheral nervous system which can promote neuronal regeneration by at least three routes: (i) an increase in cell surface adhesion molecular synthesis, (ii) production of a basement membrane which consists of ECM proteins, and (iii) production of neurotrophic factors and their corresponding receptors 15,16]. Therefore, an ideal scaffold onto which SCs attach, proliferate, and migrate plays a key role in neural tissue engineering.

Several biomaterials have been investigated for neural tissue engineering 1,16–18]. Among these, poly (hydroxyalkanoates) (PHAs) are a family of biological polyesters produced by microorganisms as intracellular carbon and energy sources. The physical and chemical properties of PHAs can be controlled by changing the monomer composition 19]. Poly (3-hydroxybutyrate) (PHB) and its copolymer with 3-hydroxyvalerate (PHBV) are two among the most common members of PHAs that proved to possess favorable physicochemical and biological properties and have, thus, found increasing applications in the fabrication of tissue engineering scaffolds 17,20]. PHB is known as a rigid and highly crystalline polymer with slow degradation rate that results in a poor processing window and higher cost, while PHBV is more flexible and easier to process than PHB as a result of its lower glass transition and melting temperatures 21,22].

Blending of PHB with PHBV results in a decrease of the melting temperature, leading to the possibility to process the materials at lower temperature in order to form specific anatomical shapes while avoiding or limiting the degradation23], Thus, PHBV can be blended with PHB to obtain a scaffold with improved physical and mechanical characteristics, as well as cell adhesion, proliferation and degradation properties.

Electrospinning of PHB(50)/PHBV(50) blends as scaffolds for bone tissue engineering were reported by Sombatmankhong *et al*. 24,25]. The 50/50 wt PHBV/PHB mats showed no cytotoxicity to human osteoblasts and the highest alkaline phosphatase activity in comparison to those of neat PHB and PHBV scaffolds. Zonari *et al.* 5] also reported that PHB(30)/PHBV(70) nanofibrous scaffolds can be used as a substrate for endothelial differentiation of hASCs and vascularization of bone tissue.

PHB and PHBV scaffolds have also shown a progressive potential for use in neural tissue engineering as a substrate for SCs culture. For instance, Sangsanoh *et al*. 18] and Swantong *et al.* 26] investigated the *in vitro* response of SCs on various types of electrospun nanofibers including neat PHB and PHBV and confirmed that these materials are non-toxic to cells. *In vitro* studies have also shown increased proliferation of SCs on PHB microfibrous conduits coated with ECM molecules 27,28]. *In vivo* studies proved that PHB conduits seeded with SCs could bridge nerve gaps in both spinal cord and peripheral nerves 27,29]. Despite both PHB and PHBV having hydrophobic surfaces and lacking functional groups, their support of limited cell adhesion and differentiation still shows promise for the field of neural tissue engineering 30]. Blending synthetic and natural polymers is one of the most effective methods for providing scaffolds with improved cell adhesion, bioactivity, and degradation rate for tissue engineering applications 9]. So far, many attempts have been made to design and develop appropriate electrospun nanofibrous scaffolds for neural tissue engineering by solution blending of synthetic biopolymers with natural polymers such as hyaluronic acid 31,32], gelatine 9], collagen 2,31,32] and chitosan 13]. It is well known that collagen is the major element of natural ECM, induces low immune response, is a good substrate for cell adhesion, and can be remodelled 32,33]. Therefore, collagen is an optimal candidate among ECM proteins to be blended with PHAs to obtain a scaffold that can enhance cell adhesion and differentiation.

Despite several studies which have aimed at finding an optimal biodegradable three-dimensional scaffold for re-myelination and aid in nerve regeneration, PHB/PHBV blended nanofibrous scaffolds displaying bioactive cues have not been investigated in depth. Hence, electrospun PHB/PHBV random and aligned nanofibers were fabricated in the present study in order to optimize the effect of polymer composition and nanofiber alignment on SCs adhesion, proliferation, and differentiation. The further effect of collagen motifs on cell activity was also assessed by fabricating blended PHB/PHBV/collagen type I aligned nanofibrous scaffolds.

## Results

### Scaffolds Characterization

**Figure 1:**
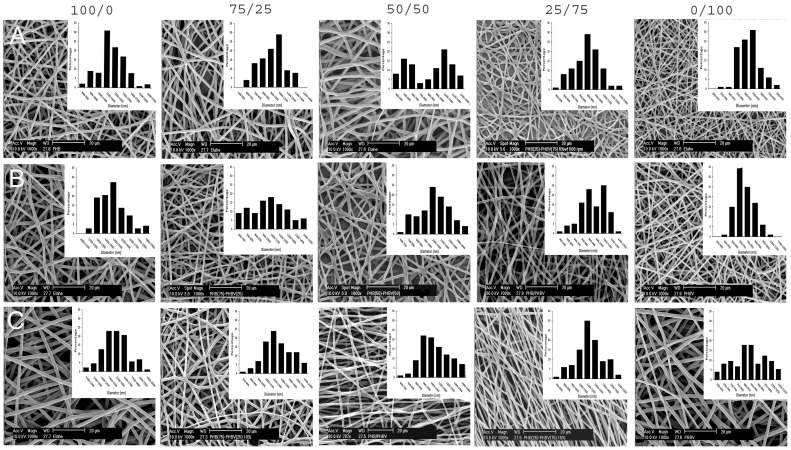
SEM images and histograms illustrating the diameter distribution of PHB/PHBV nanofibers. Polymer concentrations of (A) 6, (B) 8 and (C) 10% wt/wt were used. The voltage, flow rate and collecting conditions were fixed at 16 kV, 1.5 ml/h and 15 cm, respectively. Scale bars represent 2 0 µm for SEM images. Y axis range is 0–35% for all histograms. X axis ranges are 800–1700 nm (A 100/0), 500–1400 nm (A 75/25), 400–1300 nm (A 50/50), 400–1300 nm (A 25/75), 200–650 nm (A 0/100), 1000–1900 nm (B 100/0), 600–1500 nm (B 75/25), 600–1500 nm (B 50/50), 400–1300 nm (B 25/75), 300–1200 nm (B 0/100), 1100–2000 nm (C 100/0), 700–1600 nm (C 75/25), 600–1500 nm (C 50/50), 700–1600 nm (C 25/75) and 600–1100 nm (C 0/100).

SEM images of the surface morphology, microstructure of the fabricated scaffolds and diameter distribution of nanofibers are shown in Figure 1. Briefly, all five PHB/PHBV blend compositions were successfully electrospun at concentration of 6, 8 and 10% wt. The variation of fiber diameter with blend ratio and polymer concentration is shown in Figure S1 (supplementary information). For a fixed voltage, flow rate and collecting conditions, a decrease in polymer concentration is expected to decrease the fiber diameter, which was statistically significant for PHB(100)/PHBV(0), PHB(50)/PHBV(50) and PHB(0)/PHB(100) blends. For the PHB(75)/PHBV(25) blend, diameter differences were statistically significant by decreasing concentration from 8% to 6% and for PHB(25)/PHBV(75) from 10% to 8% concentration. With increasing the ratio of PHBV in the blends, a decrease of the fiber diameter was observed at all polymer concentrations. For a fixed concentration and composition, maximum ranges of fiber diameter were seen at a concentration of 6% in case of PHB(0)/PHBV(100). When the PHBV ratio increased, the fiber diameter changed from 1208±149 nm to 451±65 nm, while keeping the polymer concentration at 6% wt. Similarly, when the polymer concentration increased, the fiber diameter changed from 451±65 nm to 853±126 nm while keeping the polymer composition constant.

**Figure 2:**
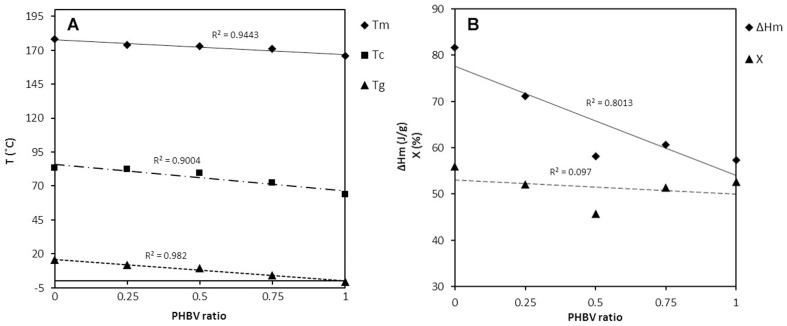
Thermal properties of PHB/PHBV nanofibers versus blend compositions. (A) melting temperatures (T_m_), glass transition temperature (T_g_) and crystallization temperatures (T_c_); (B) melting enthalpy (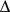
H_m_) and crystallization percentage (χ).

The miscibility of blend components was analyzed by evaluating the changes in the T_m_ and in the crystallization percentage as a function of composition ratio using DSC. The temperature range chosen for the heating step was from −50°C to 200°C to cover the melting and crystallization processes of the PHB/PHBV blends. The trends obtained from first heating and cooling scans are presented in Figure 2. By increasing the amount of PHBV, T_m_ decreased from 178.96°C to 166°C, Tg from 15.513°C to −0.565°C and T_c_ from 83.51°C to 63.86°C. Similarly, ΔH_m_ decreased from 81.65 J/g to 57.35 J/g and χ from 74.91% to 39.28%.

**Figure 3:**
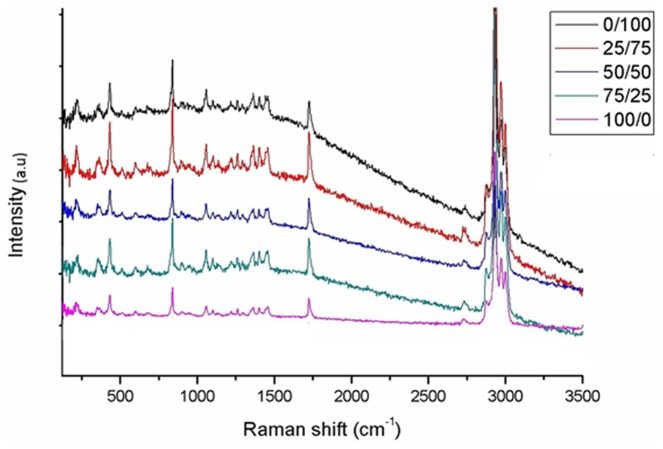
Raman spectra of PHB/PHBV nanofibers in different blend compositions.

Raman spectra of pure PHB, PHBV and their blends together with main Raman shifts and assignments of the Raman bands of PHB/PHBV samples are shown in Figure 3 and Table S2. The Raman spectra of different blends are similar to each other, because of the structural resemblance of PHB and PHBV. As a result of their different crystallinity degrees, different scaffold compositions presented distinct full width at half maximum at 1725 cm^−1^.

**Figure 4:**
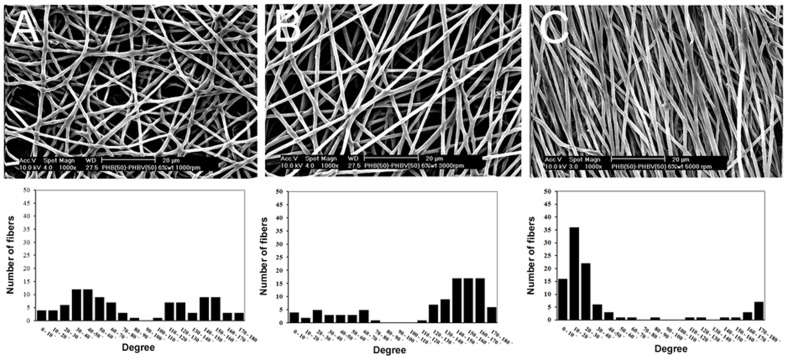
SEM images and histograms illustrating the orientation of PHB(50)/PHBV(50) nanofibrous mats. Nanofibers collected with speed of (A) 1000 rpm, (B) 3000 rpm, (C) 5000 rpm. The voltage, flow rate and collecting conditions were fixed at 16 kV, 1.5 ml/h and 15 cm, respectively. Scale bars represent 20 µm for SEM images.

To analyse the effect of speed of mandrel rotation on fiber alignment, the PHB(50)/PHBV(50) blend was selected, after also considering the analysed mechanical properties and initial cell response. The morphology of aligned and randomly oriented PHB(50)/PHBV(50) nanofibers was examined by SEM. As shown in Figure 4, the degree of fiber alignment was evaluated as the number of fibers oriented in a determined direction with respect to a horizontal base line. In the case of random nanofibers, the broad distribution of fiber angles indicates a random orientation. Conversely, aligned nanofibers displayed a narrow distribution of fiber orientation. Briefly, the distribution of fiber angles was narrower and narrower with increasing the speed of the collector drum from 1000 to 5000 rpm. Average fiber diameters of aligned and randomly oriented nanofibers collected with speeds of 1000, 3000 and 5000 rpm were 963±117 nm, 986±112 nm, and 925±156 nm, respectively.

As shown in Figure S2 (supplementary information) and Table 1, the mechanical properties of the randomly oriented and aligned PHB/PHBV nanofibers differed considerably. While the percent of elongation of aligned nanofibers is approximately ten times more than randomly oriented nanofibers, the young modulus and tensile strength are lower than that of random mats. As expected, the mechanical properties of PHB(50)/PHBV(50) random nanofibers were between the mechanical properties of PHB(100)/PHBV(0) and PHB(0)/PHBV(100) random nanofibers.

**Table 1:** Mechanical properties of PHB, PHBV and PHB(50)/PHBV(50) random and uniaxially oriented nanofibers.

	Tensile stress (MPa)	Tensile strain (%)	Tensile modulus (MPa)	Thickness (mm)
PHB random nanofibers	1.7±0.3	5.3±0.7	67.8±23.5	0.31±0.02
PHB/PHBV random nanofibers	3.4±0.3	8.6±2.0	59.3±11.3	0.26±0.02
PHBV random nanofibers	4.9±0.5	46.1±20.0	254.0±25.4	0.19±0.01
PHB/PHBV aligned nanofibers	1.3±0.1	91.7±23.6	45.4±4.7	0.25±0.01

The surface wettability of different PHB(50)/PHBV(50) scaffolds was investigated through a water contact angle method. The results showed that the water contact angle values were 123.5±6.3° for PHB(50)/PHBV(50) random nanofibers and 107.4±5.0° for PHB(50)/PHBV(50) aligned nanofibrous scaffolds, which further decreased to 94.7±9.4° for PHB(45)/PHBV(45)/Col(10) blend nanofibers.

### Schwann cell activity on PHB/PHBV solution blended random nanofibers

Figure S3 (supplementary information) shows SEM analysis of SCs spreading and morphology on the surface of different PHB/PHBV nanofibrous scaffolds after cell seeding for 1 and 7 days. A normal bipolar extensions and spindle-shaped morphology of SCs on PHB/PHBV nanofibrous scaffolds was observed. At day 1, SCs located on the surface of the scaffolds were still rounded, while after 7 days cells have expanded and proliferated considerably.

Alamar blue and DNA quantification assays were performed during the 14 days of cell culture to investigate the metabolic activity and proliferation of SCs, respectively. As shown in Figure S4A (supplementary information) the metabolic activity of cells in all PHB/PHBV blended nanofibrous scaffolds increased with time, except at day 14 where a plateau was reached. On day 1, the metabolic activity of PHB(25)/PHBV(75) was significantly higher than that of the other samples. With increasing culturing time, all samples were rather similar except PHB (75)/PHBV (25) which showed less metabolic activity in comparison to the other blends. Between 1 and 3 days after cell seeding, the largest increase in the metabolic activity of cells was observed on the PHB(100)/PHBV(0) and PHB(50)/PHBV(50) nanofibrous scaffolds, while between day 3 and day 7 the increase of metabolic activity measured on PHBV(75)/PHB(25) composition was the largest observed.

DNA amount increased for each scaffold composition from day 1 to day 14 (Figure S4B, supplementary information), indicating SCs proliferation during interval time. The largest increase in DNA amount was observed on the PHB (100)/PHBV (0) scaffolds as well as PHB(50)/PHBV(50) composition. On day 14 the amount of DNA for both PHB (100)/PHBV (0) and PHB(50)/PHBV(50) samples was significantly more than that of other compositions.

### Schwann cell activity on PHB/PHBV and PHB/PHBV/Col aligned or random nanofibers

**Figure 5:**
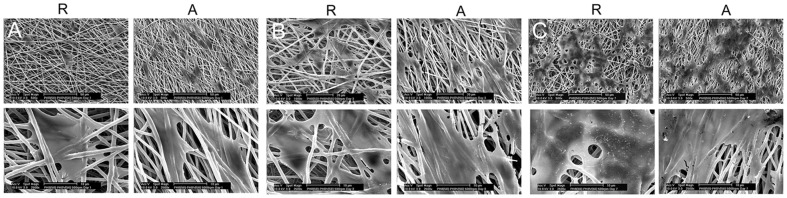
SEM images of SCs on PHB(50)/PHBV(50) random (R) and aligned (A) nanofibers. (A) 1 day, (B) 3 days, and (C) 7 days after cell seeding. Scale bars represent 50 µm for top and 10 µm for button pictures, respectively.

**Figure 6:**
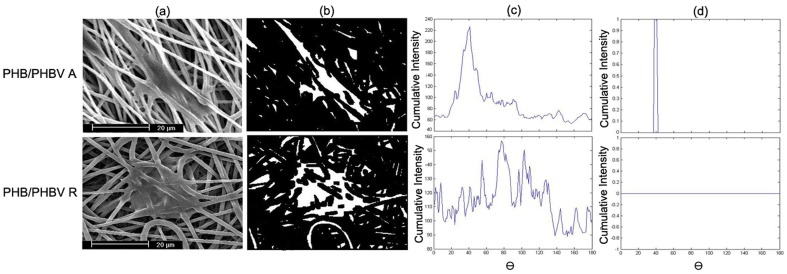
Image processing algorithms on SEM images of cultured SCs on PHB(50)/PHBV(50) random (PHB/PHBV R group) and PHB(50)/PHBV(50) aligned (PHB/PHBV A group) nanofibrous scaffolds. (A) Original SEM image of SCs on scaffold after 1 day of cell culture; (B) binary image showing the processing output of nonlinear diffusion filtering; (C) Radon transform results of binary image for a range of [0,180°] and with a resolution of 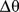
θ = 1°; (D) dominant peak of the maximum of Radon transform matrix.

Figure 5 shows SEM analysis of SCs spreading and morphology on the surface of randomly oriented and aligned PHB(50)/PHBV(50) nanofibrous scaffolds after cell seeding for 1, 3, and 7 days. These SEM images also showed normal bipolar and tripolar extensions and spindle shaped morphology of SCs on both nanofibrous scaffolds. Aligned nanofibers showed cells oriented along the direction of fibers and clustered around the aligned fibers in a longitudinal fashion, while the random fibers showed cells oriented in different directions. Figure 6 shows outputs of digital image processing analysis to investigate alignment of cultured SCs on uniaxially and randomly oriented nanofibrous substrates. A dominant peak around 40° with cumulative intensity of 1 for Radon transform output of aligned nanofibers was observed, while in the case of random substrates this dominant peak was not seen and its cumulative intensity was zero.

**Figure 7:**
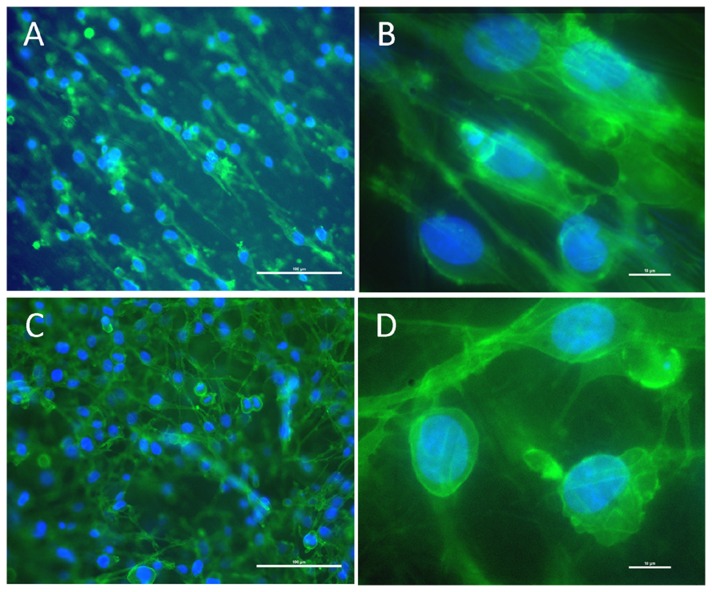
Immunostaining identification of adult rat SCs by p75LNGFR and counterstained by DAPI. Cells on aligned (A and B) and random nanofibers (C and D) showed p75LNGFR positive staining after 7 days of culture. Scale bars show 20×(100 µm) for A and C, and 100×(10 µm) for B and D pictures.

SCs functionality was also identified with p75LNGFR staining, which is one of the most common cell markers for SCs 15]. Immunocytochemistry indicated that cultured SCs were also positive for p75LNGFR, thus confirming normal cell functionality (Figure 7). Furthermore, fluorescent miscroscopy also confirmed spread cell morphology and tight attachment to the electrospun mats in addition to orientation of SCs in the direction of the fiber alignment, similar to what was observed by SEM and image processing algorithms.

### Metabolic Activity and Proliferation of SCs on Scaffolds

**Figure 8:**
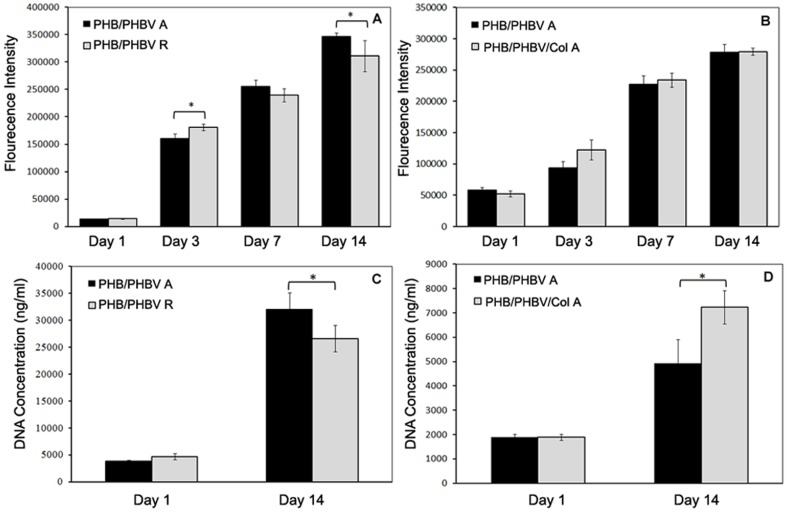
Alamar blue and DNA quantification assay results to compare metabolic activity and proliferation of SCs. (A, C) PHB(50)/PHBV(50) random nanofibers (PHB/PHBV R group) and PHB(50)/PHBV(50) aligned nanofibers (PHB/PHBV A group); (B, D) PHB(50)/PHBV(50) aligned nanofibers (PHB/PHBV A group) and PHB(45)/PHBV(45)/collagen(10) aligned nanofibers (PHB/PHBV/Col group) during 14 days of culture. Asterisks represent significant difference at *p*≤0.05.

SCs were cultured for 14 days on PHB/PHBV random and aligned nanofibers to evaluate the influence of scaffold topography on cell metabolic activity and proliferation. SCs metabolic activity increased with time in both scaffolds. While metabolic activity of SCs on random nanofibers was significantly higher than on aligned fibers at day 3, this trend was reversed by day 14 (Figure 8A). DNA amounts (Figure 8C) increased for each scaffold from day 1 to day 14. After 2 weeks a significant difference between cell proliferation on aligned and randomly oriented nanofibers was measured.

Similarly, Figures 8B and 8D present the effect of collagen incorporation on metabolic activity and proliferation of SCs. No significant difference between metabolic activity of SCs on PHB/PHBV/Col and PHB/PHBV aligned nanofibers was observed, while proliferation of SCs on PHB/PHBV/Col scaffold was significantly higher than PHB/PHBV aligned nanofibrous scaffolds at day 14.

**Figure 9:**
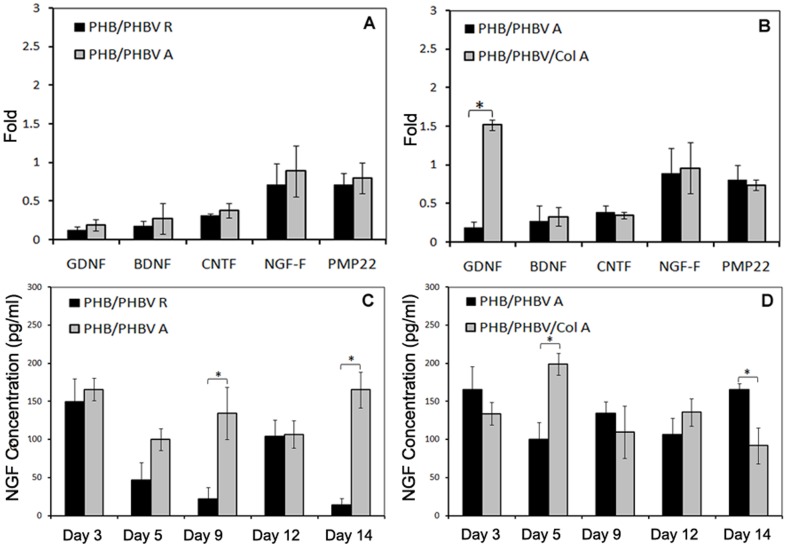
Real-time PCR analysis and NGF assay results to compare gene expression and secretions of NGF released from SCs. (A, C) PHB(50)/PHBV(50) random nanofibers (PHB/PHBV R group) and PHB(50)/PHBV(50) aligned nanofibers (PHB/PHBV A group); (B, D) PHB(50)/PHBV(50) aligned nanofibers (PHB/PHBV A group) and PHB(45)/PHBV(45)/collagen(10) aligned nanofibers (PHB/PHBV/Col group) during 14 days of culture. Asterisks represent significant difference at *p*≤0.05.

The gene expression of different SCs markers was analysed using quantitative RT-PCR (Figure 9A). No statistically significant difference was observed on SCs cultured on these groups for GDNF, BDNF, CNTF, NGF-F and PMP22, while the presence of collagen in the scaffolds resulted in a significant up-regulation of GDNF expression (Figure 9B) in comparison to aligned nanofibers in the absence of collagen, indicating that collagen might have accelerated SCs differentiation.

To further analyse cell differentiation on these scaffolds, we measured the amount of NGF secreted by the cells. There was no significant difference between NGF released from SCs on different groups at days 3, 6 and 12, whereas aligned nanofibers supported a statistically higher production of NGF at days 9 and 14 in comparison to random nanofibers (Figure 9C) (p≤0.05). Collagen containing scaffolds exhibited the highest concentration of secreted NGF at day 6 compared to unmodified aligned PHB(50)/PHBV(50) nanofibers, while after 14 days of culture the concentration of NGF on collagen containing nanofibers was significantly lower than unmodified PHB/PHBV scaffolds (Figure 9D).

## Discussion

### Characterization of Scaffolds

As previously mentioned, the use of artificial polymeric scaffolds to prepare a suitable environment for nerve regeneration has become a promising technique for the restoration of major nerve injuries 1]. To achieve this goal, mimicking the architecture of ECM is one of the critical properties of tissue engineering scaffold 35]. 36]. It is generally accepted that SCs play a crucial role during nerve regeneration through the production of neurotrophic factors, formation of myelin sheath and extraction of extracellular proteins, as well as acting as a physical guide for the newly regenerating axons 18,28]. Recently, there has been interest in the use of polymeric substrates that are pre-seeded with SCs to act as a persistent source of neurotrophic factors and an effective scaffold for enhancing nerve regeneration [14], [23]. PHB and PHBV polymers have been experimentally used as an alternative to direct epineural repair and bridge short and long-gap nerve injury models. In this study, the potential of PHB/PHBV and PHB/PHBV/collagen electrospun nanofiber blends pre-seeded with SCs has been investigated. Normally, the effects of different processing parameters in electrospinning should be assessed for individual polymers and solution blends. Hereby, electrospinning of various solution blend compositions of PHB/PHBV (100/0, 75/25, 50/50, 25/75 and 0/100) was successfully used in three concentrations of 6%, 8% and 10 % wt/wt for the generation of nanofibrous scaffolds (Figure 1). Control of fiber diameter was possible by changing the polymer concentration and blend composition (Figure S1, supplementary information). The fiber diameter is expected to increase with increasing polymer concentration due to an increase in the viscosity and surface tension of polymers solution as also demonstrated by other researchers 37–40]. Additionally, fiber diameter decreased by increasing the amount of PHBV in the solution blend, probably due to a consequent decrease in the viscosity of polymer solution as a result of the lower molecular weight of PHBV.

The homogeneity of the polymeric blends is important in terms of their internal structural and mechanical integrity 41]. Therefore, DSC and Raman spectroscopy were used to investigate the thermal and physical properties of blended polymeric nanofibers. The curves shown in Figure 2 revealed that the addition of PHBV to PHB, even in quantities as small as 25% in mass, decreased glass transition, melting temperature and crystallinity of the resulting nanofibers as well as broadened the processing window, since there was improved melt stability at lower processing temperatures, similar to the results obtained by other researchers with the same polymers 34,42–44]. Evidently, good flexibility is an important factor for a nerve tissue-engineered conduit to resist tearing and stretching forces, and retain stable shape during the regeneration process. The influence of PHBV content on thermal behavior of PHB/PHBV blends proves that the mixture of hydroxybutyrate with hydroxyvalerate is less crystalline, not as fragile, more flexible and more readily processable than PHB itself 45].

The Raman spectra of PHB/PHBV electrospun scaffolds indicated that the band centered at 1725 cm^−1^ for different samples depicted distinct full width at half maximum: PHB(100)/PHBV(0) (30 cm^−1^), PHB(75)/PHBV(25) (40 cm^−1^), PHB(50)/PHBV(50) (44 cm^−1^), PHB(25)/PHBV(75) (46 cm^−1^) and PHB(0)/PHBV(100) (52 cm^−1^). The difference between band widths can be assigned to the different crystallinity of these two polymers. These results are in agreement with those obtained from DSC, and the study of Izumi *et al*. 46] showing that crystallinity degree of PHBV decreased as the hydroxyvalerate (HV) content increased. Despite similarities among PHB and PHBV Raman spectra, the relative intensity of the bands at some wave lengths is different. However, it is not possible to correlate the changes of these bands with the respective blend composition, since the samples have different crystallinity that may influence the Raman intensity of these bands.

It has well been established that fiber orientation influences cell adhesion and growth, and guides cellular elongation 13]. In this study, the influence of the drum rotating speed on orientation of nanofibrous webs was examined to design aligned PHB/PHBV nanofibrous scaffolds. The distribution of fiber angles was narrower in proportion to the increase of rotating speed, thus resulting in improvement of fiber alignment (Figure 4). These results indicate that the higher speed of rotation of the drum collector exerts a larger tangential force on the polymer solution jet, inducing alignment of fibers without considerable influence on the fiber diameter. Similar effect was also observed by Wang *et al.* and Cooper *et al.* during the fabrication of chitosan and chitosan-PCL nanofibers, respectively 13,47].

Also, an ideal nerve conduit should have a Young's modulus approaching that of nerve tissues to withstand manual manipulation, *in vivo* physiological loading during nerve regeneration, and retain its structure after implantation 48]. It is generally accepted that Young's modulus of a rabbit tibial nerve and a rat sciatic nerve in the longitudinal direction, for example, is in the range of 0.50 and 2.72 Mpa, respectively 13,48].

As shown in Table 1, the tensile strength of PHB(50)/PHBV(50) random and aligned nanofibrous scaffolds were 337±0.3 and 1.30±0.1 MPa, respectively. Moreover, the percent elongation of aligned fibrous scaffolds was considerably higher in comparison to the randomly oriented scaffolds which indicated their good elasticity and flexibility. The difference between mechanical properties of random and aligned nanofibers is probably caused by different breaking mechanisms. We can hypothesize that the increased strength of random fibers results from more compactness of nanofibers in the web, which can assist them to withstand higher loadings. Another interesting result was observed when comparing the mechanical properties of unmodified PHB and PHBV nanofibers. Surprisingly, mechanical properties (tensile strength, tensile modulus and elongation percentage) of PHBV and PHB(50)/PHBV(50) nanofibrous mats were considerably higher than that of PHB fibers. Many studies have confirmed that PHB copolymers with 3-hydroxyvalerate (PHBV) are more flexible and more easily processed than PHB as a result of its lower glass transition and melting temperatures 21,22], which is in agreement with our DSC results (Figure 2).

In this study, we fabricated five different composite of PHB/PHBV solution blending electrospun nanofibers for nerve tissue engineering and investigated physico-thermal properties of all solution blends. In conclusion, PHBV copolymer partly fills the gap of toughness and flexibility of PHB; however, PHB/PHBV composite nanofibrous mats exhibit lower melting points with respect to PHB, narrowing the utilization temperature range.

### Interaction between SCs and Scaffolds

Although the *in vitro* assays illustrated the cellular biocompatibility of the fabricated scaffolds, direct cellular interactions with the material surface also plays an important role in tissue regeneration. Effective attachment of SCs onto scaffolds is a critical step for the fabrication of tissue engineered nerve conduits. For example, SCs morphology and directionality are two key factors contributing to neurogenesis 13]. SEM analysis showed physiological cell morphology in PHB/PHBV blended samples in addition to increased proliferation between day 1 and day 7 (Figure S3, supplementary information). Bi-polar and tri-polar extensions with spindle shaped morphology are characteristics of normal SCs 14], also observed in SEM images of day 1. The high surface area and extracellular-like physical environment provided by nanofibers compared to that of other non-fibrillar surfaces may have led to an increase in cellular attachment and the observed cell polarity. For instance, Leong *et al*. 49] indicated that the cell-matrix adhesion in nanofibrous mats is stronger due to an increase in the surface area of the nanofibrous mats and their three-dimensional features. Similar results were also observed by Sangsanoh *et al*. 18] on different polymeric nanofibers composed of neat PHB, PHBV, polycaprolactone (PCL), poly(L-lactic acid) (PLLA), and chitosan seeded with SCs.

Aligned fibrous scaffolds could exhibit SCs columns (Figure 5), also known as bands of Büngner. Bands of Büngner are formed when SCs proliferate and the remaining connective tissue basement membrane lines endoneurial tubes 50,51].

Indeed, bands of Büngner comprise longitudinally aligned SCs strands that guide selectively regrowing axons.13,50]. Wang *et al.* have suggested that a presumed mechanism for the formation of band of Bunger is the polarized expression of adhesion proteins such as dystronlycan and α6β4 integrin on the SCs axis 47]. It can also be expected that SCs alignment obtained on the oriented nanofibers might induce oriented axonal growth as a result of classical contact guidance 14]. Similar results have been observed by different researchers, who have also reported oriented arrangement of SCs on aligned electrospun nanofibrous mats 13,14,47,52].

In addition to ordinary SEM imaging, we made use of digital image processing algorithms to evaluate the orientation of cultured SCs on aligned and random fibrous scaffolds. After binarization and calculation of Radon transform, it was possible to determine the main orientation angle of cultured SCs. Results confirmed cell orientation on aligned fibers with a high resolution and an accuracy of 95%.

Immunostaining with p75 low-affinity NGF receptor (p75LNGFR) marker also showed normal topography and activity of SCs on aligned nanofibrous scaffolds (Figure 7). p75LNGFR is one of the two receptor types for neurotrophins, a family of protein growth factors that stimulate neuronal cells to survive and differentiate. It is well known that SCs in the distal stump of injured peripheral nerves synthesize the p75LNGFR receptor. You *et al*, 53] showed that the presence of p75 protein in SCs is necessary for reinnervation and the expression of p75 by SCs provides a suitable environment for the regenerating axons, especially in the early stages of regeneration. Zorick and Lemke showed the effective role of LNGFR in all stages of SCs differentiation and myelination. Here, we can hypothesize that the positive staining of cultured SCs on the designed blended scaffolds indicates SCs maintain their potential to support axonal regeneration 54].

The next step to consider PHB/PHBV nanofibers in nerve tissue engineering was to ensure that attached SCs could proliferate and perform their normal functions. To evaluate the influence of blend composition on SCs metabolic activity and cell proliferation, SCs were cultured for 14 days on PHB/PHBV random nanofibers. As shown in Figure S4B (supplementary information), proliferation of SCs increased within 14 days of culture and the amount of proliferation for PHB(100)/PHBV(0) and PHB(50)/PHBV(50) compositions was higher than that of other samples. Likewise, Suwantong *et al*. [15] showed that SCs can proliferate on PHB or PHBV nanofibers within 5 days. As previously mentioned, experimental evidence indicates that grafts seeded with SCs prior to implantation have shown enhanced nerve regeneration *in vitro* and *in vivo* 55]. For example, Yu *et al*. 56] successfully used collagen/PCL nanofibers seeded with SCs to regrow axons. Schnell *et al*. 57] also indicated that electrospun collagen/PCL substrates pre-seeded with SCs can support oriented neurite outgrowth and glial migration from dorsal root ganglia explants.

To further evaluate the synergistic effect of alignment with bioactive motifs from the basement membrane, we cultured SCs aligned electrospun PHB(50)/PHBV(50) nanofibers with or without blending with collagen type I. In general, aligned nanofibers enhanced SCs proliferation on day 14, and metabolic activity on days 3 and 14, in comparison to random fibers. Gupta *et al*. 14] believed that, rough surface topography and more interconnected pores make random nanofibers better scaffolds for SCs proliferation. Our morphological studies showed that the direction of SCs is parallel to the direction of fiber alignment. Therefore, it can be concluded that the arrangement of cells in controlled architecture has beneficial effects on SCs metabolic activity.

Incorporation of collagen onto PHB(50)/PHBV(50) electrospinning solution did not result in any significant increase of SCs metabolic activity compared to unmodified nanofibrous scaffolds (Figure 8B), while a significant increase in SCs proliferation was observed (Figure 8D). The improved proliferation observed on collagen blended nanofibrous scaffolds could be firstly explained as a result of increased hydrophilicity, which increases serum proteins adsorption. Secondly, the presence of collagen molecules on the surface of nanofibers could be associated to a preferential adhesion of SCs to these protein motifs, better mimicking their native environment. Previous studies also confirmed our results indicating increased cells proliferation and attachment on collagen functionalized scaffolds. For example, Meng *et al.* reported that the PHBV/collagen nanofibrous scaffolds accelerated the adhesion and growth of NIH-3T3 cells more effectively than PHBV nanofibrous scaffolds 45]. Enhanced attachment and viability of neural stem cell on nanofibrous collagen immobilized scaffolds was also reported by Li *et al*. 58].

Previous studies have shown that the terminal chemical groups present on the surface of scaffolds could control cell growth and differentiation 6]. Wang *et al*. 59] believed that a fraction of N-containing groups such as amine introduced to the surface may be positively charged at physiological pH because of protonation in the culture medium, which helps the adhesion of cells that carry negative charges on the membrane surface. Ren *et al*. 60] also showed that amino groups are more effective than hydroxyl and carboxyl groups towards nerve stem cell proliferation and migration. Hence, it can be concluded that the presence of amino groups on the surface of PHB/PHBV/Col nanofibrous scaffolds and the higher efficiency of amino groups with respect to hydroxyl and carboxyl groups may account for the observed enhancement in SCs proliferation.

Once axonal growth is initiated, cell survival and continued axonal regeneration depend on the supply of trophic physiological growth factors, which are synthesized by SCs 61,62]. The expression of these cytokines changes during SCs differentiation. PMP22 and CNTF expression increase during myelination, while low levels of BDNF are present in SCs of either developing or mature nerves 63]. Therefore, real-time PCR analysis of the genetic expression of these neural cytokines gives more information about SCs maturity and differentiation on the designed scaffolds. In this study, there was no significant difference between all gene expressions of random and aligned PHB/PHBV nanofibrous scaffolds, suggesting substrate orientation has not increased SCs differentiation and myelination (Figure 9A). With incorporation of collagen in the scaffolds, GDNF expression of SCs on PHB/PHBV/Col aligned scaffolds significantly increased compared to neat nanofibers (Figure 9B). GDNF is an important growth promoting factors for dopaminergic and motor neurons, especially in the case of chronic differentiation 62]. Li *et al*. 64] showed that increased expression of GDNF by genetically modified SCs improved peripheral nerve regeneration. Piirso *et al*. suggested that GDNF promotes myelination of small caliber axons that normally do not myelinate and enhances myelination in neuron-SCs co-cultures 65]. In this study, high expression of GDNF factor on collagen containing scaffolds suggests a possible positive role of collagen in SCs differentiation.

Another important neurocytokin for SCs differentiation and myelination is NGF. It has been proved that NGF influences myelination of axons by both SCs and oligodendrocytes 65,66]. Indeed SCs are commonly recognized as the major source of NGF synthesis in the axotomized nerve 67]. In this study, ELISA assay results of NGF secretion showed significant increase of NGF released by SCs cultured on aligned nanofibers on days 9 and 14 in comparison to that of random mats (Figure 9C). Collagen containing scaffolds also showed significantly more NGF secretion of SCs in comparison to that of PHB(50)/PHBV(50) scaffolds on day 6. At day 14, NGF secretion was, however, significantly lower on collagen containing scaffolds than on PHB/PHBV ones (Figure 9D). This might be explained by a faster differentiation of SCs when cultured in presence of collagen, thereby reducing the production of NGF at later culture times.

Therefore, PHB/PHBV and PHB/PHBV/Col aligned nanofibrous scaffolds fulfil the requirement of being a stable mechanical and physical support for axonal growth, whereas the seeded SCs produce the required neurotrophic factors, which are helpful for stimulating the outgrowth of axons. Further studies should aim at deepening our understanding whether this composition could also be optimal for SCs differentiation and myelinic membrane formation during extended culturing periods and in preclinical animal models.

## Materials and Methods

### Materials

Poly(R-3-hydroxybutyrate) (PHB), M_w_ = 437000, Poly(3-hydroxybutyrate)-co-(R-3-hydroxyvalerate) (PHBV) with 5% wt poly (3-hydroxyvalerate), M_w_ = 150000, and solvents (chloroform and N, N-dimethyl formamide (DMF) and Hexaflouroisopropanol (HFIP)) were purchased from Sigma Aldrich (St. Louis, MO, USA) and Merck Co. (Germany), respectively. Acid soluble collagen type I powder of bovine origin was a generous gift from Kensey Nash Corporation (USA, Catalogue number 20003–04). Purchased reagents for the cell culture were as follows: fetal bovine serum (FBS) from Hyclone (Logan, UT, USA), Dulbecco Modified Eagle Medium (DMEM), phosphate buffered saline (PBS), penicillin, streptomycin and trypsin-EDTA from Gibco BRL (Gaithersburg, MD, USA).

### Electrospinning

All polymeric solutions of PHB/PHBV blends were prepared and stirred overnight at 60°C before use by dissolving two components in a mixture of chloroform (90) and DMF (10) solvents at concentrations of 6, 8 and 10% wt. The desired solution was loaded into a syringe and the flow rate was controlled using a syringe pump (KDS 100, KD Scientific). The other end of the syringe was connected to a needle, on which a positive high voltage (16 kV) was applied using a high voltage generator (Gamma High Voltage Research Inc., USA). The polymeric solution was fed at a rate of 1.5 ml*min^−1^ through a 10 cc syringe with a 23 G needle placed 15 cm from a rotating mandrel collector with speed of 1000 and 5000 rpm to collect PHB/PHBV random and aligned nanofibers, respectively. Temperature and humidity were monitored during the process and ranged between 24–26 °C and 37–42%, respectively.

PHB/PHBV/Col nanofibers were also fabricated by solution blending of PHB, PHBV and collagen at a ratio of 45∶45∶10 (wt/wt), and at a total concentration of 4% (wt/wt). In short, collagen was dissolved in HFIP at room temperature, and then PHB and PHBV were added to the collagen solution. The final solution was stirred for 24 h at room temperature. The solution was electrospun with mentioned flow rate and voltage and aligned PHB/PHBV/Col nanofibers were collected with a speed of 5000 rpm.

### Scanning electron microscopy (SEM)

The morphology of electrospun fibers spun under the above mentioned conditions was observed using scanning electron microscopy (SEM) (XL 30 ESEM-FEG, Philips). Fiber diameters were calculated from SEM micrographs by measuring 100 fibers per condition using Manual Microstructure Distance Measurement software (Nahamin Pardazan Asia Co., Iran).

### Differential scanning calorimetry (DSC)

The DSC experiments were conducted in a Q100 calorimeter with refrigerated cooling system (TA Instruments). Prior to the scans, temperature and energy calibrations were performed with an indium standard. All the samples were (a) held at −50°C for 5 min, (b) heated from −50°C to 200°C, (c) held at 200°C for 5 min to erase the thermal history, (d) cooled down from 200°C to −50°C, (e) held again at −50°C for 5 min, and (f) re-heated to 200°C. The heating and cooling rate was of 10°Cmin^−1^. The glass transition temperature (T_g_), melting temperature (T_m_), crystallization temperature (T_c_) and melting enthalpy (*Δ*H_m_) of PHB/PHBV blends were determined from the heating and cooling scans. The crystallinity degree (χ) can be calculated by applying the following equations (1 and 2):
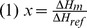


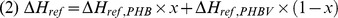



Where *ΔH_ref_* is the melting enthalpy of 100% crystalline polymer (i.e. 146 Jg^−1^ and 109 Jg^−1^ for PHB and PHBV, respectively 34]) and *x* is the weight fraction of PHB in the blend.

### Raman Spectroscopy

Confocal non-resonant Raman microspectroscopy was performed using a home-built laser-scanning Raman microspectrometer. In brief, the 647.1 nm excitation light from a Krypton ion laser was focused onto the sample through a 40x air objective (Olympus). Raman spectra were acquired in 2 s at a laser power of 35 mW under the objective. For each sample, spectra were taken at 5 randomly chosen spots and averaged. Data preprocessing was performed using routines written in MATLAB 6.5 (The MathWorks Inc., Natick, MA).

### Mechanical properties of nanofibrous scaffolds

For mechanical strength measurements, scaffolds were cut into 20×10 mm rectangular strips. Tensile test on the scaffolds was performed with a tensile testing machine (Zwick Z050, Germany) at a crosshead speed of 10 mm/min. Tensile properties were calculated from the stress-strain curves as the means of five measurements and the average value was reported with standard deviation (± SD).

### Surface wettability measurements

Water contact angle (θ) of the nanofibrous scaffolds was determined on a contact angle goniometer (Dataphysics OCA-20). Deionised water was used as a probe liquid. Water contact angle was measured by at least five independent measurements and was presented as mean ± standard deviation.

### Cell Culture

The *in vitro* experiments were performed using a rat Schwann cell line RT4-D6P2T (ATCC, USA), cultured in high glucose DMEM, and supplemented with 10% fetal bovine serum (FBS) and 1% Penicillin/Streptomycin. The cells were incubated at 37°C in a humidified atmosphere containing 5% CO_2_ and the cultured medium was changed once every 3 days. Electrospun discs with a diameter of 15 mm were soaked in 70% ethanol for 2 hr, washed twice with PBS, transferred to a non-treated 24 well plate (NUNC) and incubated overnight in basic cell culture medium. Rubber O-rings (Eriks BV, The Netherlands) were used to secure the scaffolds in place and prevent them from floating. After removing the medium, each scaffold was seeded with 50′000 cells in 50 µl basic medium and incubated for 30 min to allow cell attachment and topped up to 1 ml with culture medium.

### Cell Morphology Study

On days 1 and 7, one sample from each group was used for SEM analysis. The media were removed and the scaffolds were washed twice with PBS and fixed in 4% formalin for 30 min. After rinsing with PBS, the scaffolds were dehydrated in a series of increasing ethanol concentrations (70, 80, 90, 96, and 100%), 30 min in each concentration, before being dried using a critical point dryer (Balzers CPD-030). The samples were then sputter coated with gold (Cressington) for SEM observation (XL 30 ESEM-FEG, Philips).

### Assessment of Cell Alignment Using Digital Image Processing

To investigate cells alignment, we applied digital image processing algorithms to the SEM images of cultured SCs on scaffolds. Processing was performed on a dataset of 50 SEM images of cultured SCs on both randomly and uniaxially oriented nanofibrous scaffolds, which have been captured 1 day after cell culture and cropped to the same size of 295×235 pixels. All of the images had the same magnification of 1000x and were captured with the same brightness and contrast to ease further image analysis.

The fallowing algorithm was used to verify the probable existence of SCs orientation on fibrous scaffolds. Firstly, by Nonlinear Diffusion Filtering (NDF) the original images were smoothed. NDF preserved the high gradient objects of the image and smoothed low gradient parts of it. After that, by using binary algorithms of image processing, a binary image of the NDF output was made and Radon transform of the binary image was calculated for a range of [0,180°] and with a resolution of Δθ = 1°. After calculation of the maximum of Radon transform matrix columns and verification of a detectable dominant peak, it was possible to specify the localization of angle.

### Cell Metabolism

Cell metabolism was assessed using Alamar blue assay according to the manufacturer's protocol. Briefly, culture medium was replaced with medium containing 10% (v/v) Alamar blue solution (Biosource, Camarillo, CA, USA) and the cells were incubated at 37°C for 4 hr. Absorbance was measured at 590 nm using a Perkin Elmer Victor3 1420 Multilabel plate reader. Cell metabolism was analyzed on day 1, 3, 7 and 14 (n = 3), and medium containing Alamar blue solution was replaced with fresh medium after each measurement.

### Cell Proliferation

After 1 and 14 days of cell culture, the scaffolds were taken from the culture medium, washed in PBS and frozen at −80°C until further processing. Subsequently, they were digested at 56°C(>16 h) in a Tris-EDTA buffered solution containing Proteinase-K (1 mgml^−1^), 18.5 pepstatin A (18.5 µgml^−1^), and iodoacetamide (1 µgml^−1^) (Sigma-Aldrich).

DNA quantification assay was performed with CyQuant dye kit according to the manufacturers description (Molecular Probes, Eugene, Oregon, U.S.A.), using a spectrofluorometer (Victor3, Perkin Elmer, U.S.A.), at an excitation wavelength of 480 nm and an emission wavelength of 520 nm.

### ELISA Assay of NGF Secretion

To quantify the concentration of nerve growth factor (NGF) in cell cultured supernatant, commercially available ELISA kits were used according to the manufacturer's instruction (Promega). The plates were read at 450 nm and analysed using Elisa machine (Lightcycler II, Roche Diagnostics GmbH, Germany). Secretions of NGF were measured following 1, 3, 6, 9, 12 and 14 days of culture.

### RNA extraction and quantitative real-time RT-PCR

To analyse the expression of neural markers by SCs, total RNA was isolated using a combination of the TRIzol® method with the NucleoSpin®RNA II isolation kit (Bioké). Briefly, at day 7 of cell culture, scaffolds (n = 3) were washed with PBS once and 1 ml of TRIzol reagent (Invitrogen) was added to the samples. After five minutes, the samples were stored at −80 °C for RNA isolation. After chloroform addition and phase separation by centrifugation, the aqueous phase containing the RNA was collected, mixed with equal volume of 75% ethanol and loaded onto the RNA binding column of the kit. Subsequent steps were in accordance with the manufacturer's protocol. RNA was collected in RNAse-free water. The quality and quantity of RNA was analysed by gel electrophoresis and spectrophotometry. Seven hundred fifty nanograms of RNA were used for first strand cDNA synthesis using iScript (Bio-Rad) according to the manufacturer's protocol. One µl of undiluted cDNA was used for subsequent analysis. Time reverse transcription polymerase chain reaction (real-time PCR) was performed on an iQ5 real time PCR machine (Bio-Rad) using SYBR Green supermix (Bio-Rad). Expression of neural marker genes was normalised to β-Actin levels and fold inductions were calculated using the comparative ΔCT method. The expressions of brain derived neurotrophic factor (BDNF), glia derived neurotrophic factor (GDNF), nerve growth factor (NGF), ciliaryneurotrophic factor (CNTF), and peripheral myelin protein 22 (PMP22) were detected by the real-time PCR (Table S1).

### Immunofluorescent staining

The cells were washed in PBS and fixed with paraformaldehyde (4% v/v)(Sigma-Aldrich) in PBS for 30 min. Fixed cells were washed with PBS. Blocking was carried out in a blocking buffer that consisted of 10% w/v BSA solution in PBS (1X) for 1 h. Primary antibodies against p75 low affinity NGF receptor (p75LNGFR, 1∶500, Abcam; ab6172) were applied in dilute buffer consisting of 10% BSA in PBS at room temperature for 2 h or overnight at 4°C. Cells were then washed and the secondary antibody, goat anti-mouse IgG conjugated-fluorescein isothiocyanate (FITC, 1∶50), was applied for 45 min at 37°C. Cells were counterstained with DAPI for 10 min and observed under fluorescence microscope. For negative controls, the primary antibody was excluded.

### Statistical analysis

All data presented are expressed as mean ± standard deviation (SD). Statistical analysis was carried out using one-way analysis of variance (ANOVA) followed by a Tukey's post hoc test. A value of p<0.05 was considered statistically significant.

### Conclusion

In this study, various PHB/PHBV solution blended compositions were electrospun and the effect of blend composition on fiber diameter was studied. SEM investigations showed that the diameter of nanofibers decreased with increasing the PHBV ratio. Characterization of samples by DSC and Raman spectroscopy showed that these electrospun nanofibers are homogenous. Crystallinity and melting temperature of nanofibers decreased with increasing the PHBV ratio. PHB/PHBV nanofibers also showed better mechanical properties and flexibility in comparison to that of neat PHB nanofibers. SEM images showed that degree of orientation increased by using a higher speed rotating rotator.

When SCs were seeded on these scaffolds, bipolar cell morphology was observed. In the case of aligned fibers, SCs oriented themselves in the direction of fiber alignment probably due to contact guidance phenomenon.

The potential of these fibrous mats as nerve scaffolds was further assessed by observing the cellular activity. Aligned PHB/PHBV nanofibrous scaffolds showed higher SCs proliferation after 14 days in comparison to random nanofibers. Incorporation of collagen to PHB/PHBV also increased SCs proliferation as well as neurotrophin secretion and GDNF expression. In conclusion, aligned PHB/PHBV and PHB/PHBV/Col nanofibrous mats provide a favorable environment for Schwann cell growth and myelin sheath regeneration.

## Supporting Information

Figure S1 **Variation of fiber diameters of PHB/PHBV nanofibers with polymer concentration and blend composition.** The voltage, flow rate and collecting conditions were fixed at 16 kV, 1.5 ml/h and 15 cm, respectively. Scaffolds prepared at 6% concentration were chosen for *in vitro* cell culture experiments.
(TIF)

Figure S2 **Tensile stress-strain curves of PHB, PHBV and PHB(50)/PHBV(50) random and uniaxially oriented nanofibers.**
(TIF)

Figure S3 **SEM images of SCs on different PHB/PHBV electrospun solution blending nanofibrous scaffolds.** (A) 1 day, and (B) 7 days after cell seeding. Scale bars represent 50 µm for top and 10 µm for bottom pictures, respectively.
(TIF)

Figure S4 **Metabolic activity and proliferation of SCs on different PHB/PHBV electrospun solution blending nanofibrous scaffolds during 14 days of culture.** (A) alamar blue assay; (B) DNA quantification assay. Asterisks represent significant difference at *p*≤0.05.
(TIF)

Table S1 **The designed primers of genes for real-time PCR.**
(DOCX)

Table S2 **Raman shift (cm^−1^) and assignment of the Raman bands of PHB/PHBV nanofibers.**
(DOCX)
