# Supplementary figures and images for: Fabrication, Characterization and Cellular Compatibility of Poly(Hydroxy Alkanoate) Composite Nanofibrous Scaffolds for Nerve Tissue Engineering
Source: PLoS One. 2013 Feb 27;8(2):e57157. doi: 10.1371/journal.pone.0057157 (PMC3584130; doi:10.1371/journal.pone.0057157)

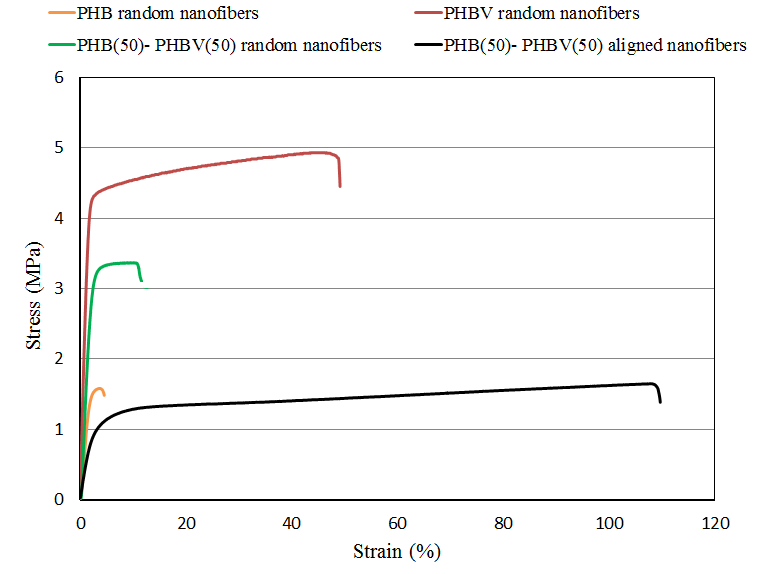

Supplement: Figure S2 — Tensile stress-strain curves of PHB, PHBV and PHB(50)/PHBV(50) random and uniaxially oriented nanofibers. (TIF) [file pone.0057157.s002.tif]

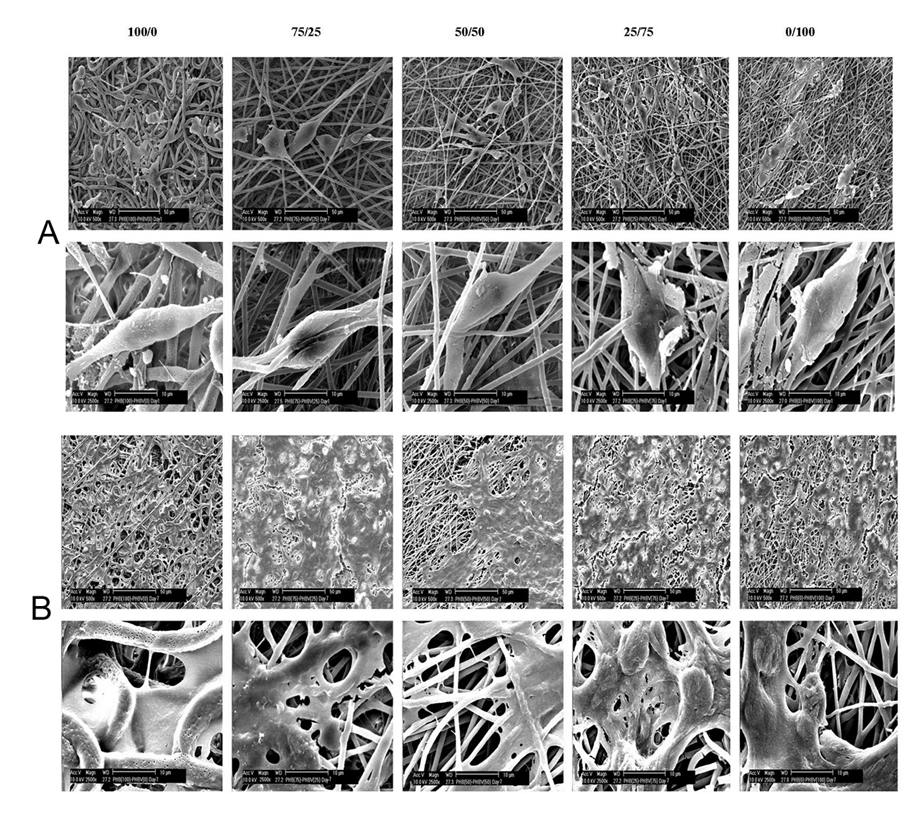

Supplement: Figure S3 — SEM images of SCs on different PHB/PHBV electrospun solution blending nanofibrous scaffolds. (A) 1 day, and (B) 7 days after cell seeding. Scale bars represent 50 µm for top and 10 µm for bottom pictures, respectively. (TIF) [file pone.0057157.s003.tif]

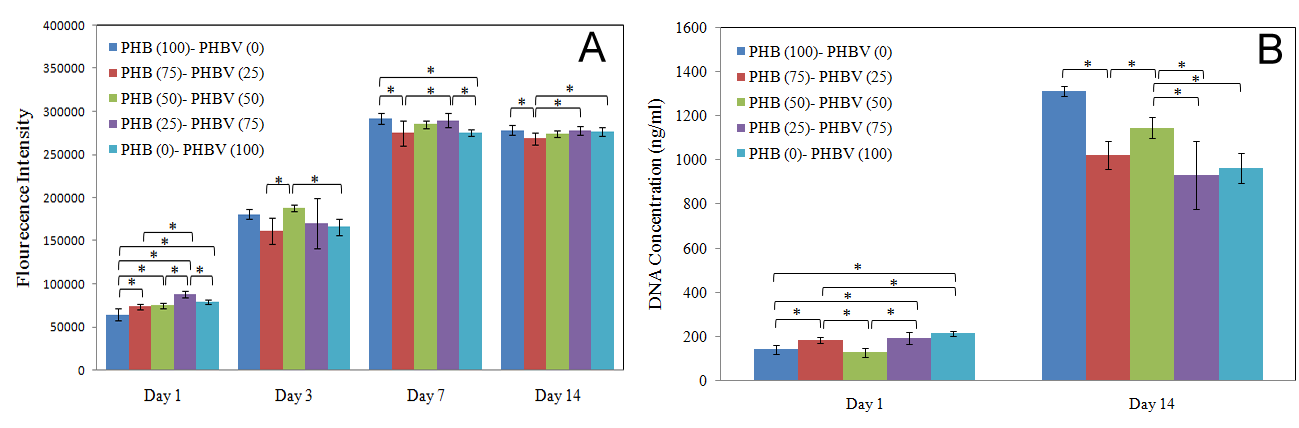

Supplement: Figure S4 — Metabolic activity and proliferation of SCs on different PHB/PHBV electrospun solution blending nanofibrous scaffolds during 14 days of culture. (A) alamar blue assay; (B) DNA quantification assay. Asterisks represent significant difference at p≤0.05. (TIF) [file pone.0057157.s004.tif]
