# Supplementary material for: Fabrication, Characterization and Cellular Compatibility of Poly(Hydroxy Alkanoate) Composite Nanofibrous Scaffolds for Nerve Tissue Engineering
Source: PLoS One. 2013 Feb 27;8(2):e57157. doi: 10.1371/journal.pone.0057157 (PMC3584130; doi:10.1371/journal.pone.0057157)
Supplement: Table S1 — The designed primers of genes for real-time PCR. (DOCX) [file pone.0057157.s005.docx]

| Gene | Primer Sequence | Length |
| --- | --- | --- |
| β-Actin | F:5’-CACCCGCGAGTACAACCTTC-3’  R:5’-CCCTATCCCACCATCACACC-3’ | 207 |
| CNTF | F:5’-TTTGCGAGAGCAAACACCTCT-3’  R:5’-TGCTAGCCAGATAGAACGGCTAC-3’ | 67 |
| GDNF | F:5’-GGCGACGGGACTCTAGAATGA-3’  R:5’-GTCAGGATAATCTTCGGGCATATTG-3’ | 194 |
| BDNF | F:5’-GCCATTCATTCAGGCTTCCA-3’  R:5’-GCCATTCATTCAGGCTTCCA-3’ | 93 |
| NGF-F | F:5’-TGATCGGCGTACAGGCAGA-3’  R:5’- GAGGGCTGTGTCAAGGGAAT -3’ | 107 |
| PMP22 | F:5’-TGTACCACATCCGCCTTGG-3’  R:5’-GAGCTGGCAGAAGAACAGGAAC-3’ | 138 |
