# Supplementary material for: Fabrication, Characterization and Cellular Compatibility of Poly(Hydroxy Alkanoate) Composite Nanofibrous Scaffolds for Nerve Tissue Engineering
Source: PLoS One. 2013 Feb 27;8(2):e57157. doi: 10.1371/journal.pone.0057157 (PMC3584130; doi:10.1371/journal.pone.0057157)
Supplement: Table S2 — Raman shift (cm−1) and assignment of the Raman bands of PHB/PHBV nanofibers. (DOCX) [file pone.0057157.s006.docx]

| **Raman shift (cm^-1^)** | **Assignment** |
| --- | --- |
| 432  841  1365  1460  1725  2929  2970 | CCO deformations  C–COO stretching  CH deformation and CH3 symmetric deformation  CH3 asymmetric deformation  C=O stretching  CH3 antisymmetric stretching  CH3 asymmetric stretching |
